# Analysis of sleep traits in knockout mice from the large-scale KOMP2 population using a non-invasive, high-throughput piezoelectric system

**DOI:** 10.1186/1471-2105-16-S15-P15

**Published:** 2015-10-23

**Authors:** Mansi Sethi, Shreyas S Joshi, Martin Striz, Neil Cole, Jennifer Ryan, Michael E Lhamon, Anuj Agarwal, Stacey J Sukoff Rizzo, James M Denegre, Robert E Braun, David W Fardo, Kevin D Donohue, Elissa J Chesler, Karen L Svenson, Bruce F O'Hara

**Affiliations:** 1 Department of Biology, University of Kentucky, Lexington, KY 40506, USA; 2 The Jackson Laboratory, Bar Harbor, ME 04609, USA; 3 Signal Solutions, LLC, Lexington, KY 40503, USA; 4 Department of Electrical & Computer Engineering, University of Kentucky, Lexington, KY 40506, USA; 5 Department of Biostatistics, University of Kentucky, Lexington, KY 40536, USA

## Background

Sleep is critical to well-being and yet the functions and regulation of sleep are poorly understood. Genomic approaches to identify genes that influence sleep may provide insight into these processes. Our current study employs a non-invasive, high-throughput piezoelectric system utilizing the breathing pattern to assess sleep-wake phenotypes in a large population of control and single-gene knockout mice; recorded as part of the Knockout Mouse Phenotype Program (KOMP2) at The Jackson Laboratory, which in turn is part of the IMPC (International Mouse Phenotyping Consortium – http://www.mousephenotype.org).

## Materials and methods

Knockout mice (15 weeks) generated on a C57BL/6NJ background[1] were phenotyped for sleep-wake parameters as part of the JAX phenotyping pipeline under 12:12 L:D, baseline conditions for 5 days using a piezoelectric system and compared to control (C57BL/6NJ) mice. The piezoelectric system consists of a sensor pad placed at the bottom of the mouse cage to record gross body movements. The pressure signals thus generated are classified by an automated classifier into sleep and wake[2,3]. The system characterizes traits that range from sleep time over 24 hours, as well as during the light and dark phase, distribution of sleep bout lengths and the breath rate during sleep periods. The system has been validated with electroencephalogram (EEG) and human observation and demonstrates a classification accuracy of over 90%[2-4].

## Results

To date, over 3500 mice representing over 180 knockout lines, and more than 1200 control mice (C57BL/6NJ) have been recorded. 15-20% of these knockout lines demonstrated altered sleep phenotypes, depending on the specific sleep traits assessed and the statistical approaches utilized. Some genes were found to specifically alter total sleep amounts or sleep bout lengths primarily in the light phase, others in the dark phase. Several genes were also found to alter breath rates during sleep. Among controls and knockouts, males sleep slightly more than females in most but not all cases. Assessment of different sets of control mice over time showed that sleep traits are consistent over days, weeks, months and years, which may contribute to the high “hit rate” we have for altered sleep phenotypes. This high hit rate may also reflect that a high percentage of genes are expressed in the brain, and that many factors alter sleep. In addition to identifying specific genes that influence sleep, correlations between different sleep traits and between sleep and non-sleep traits in these same mice may also shed light on sleep mechanisms.
